# Deep Brain Stimulation for Obesity: From a Theoretical Framework to Practical Application

**DOI:** 10.1155/2016/7971460

**Published:** 2015-12-27

**Authors:** Raj K. Nangunoori, Nestor D. Tomycz, Michael Y. Oh, Donald M. Whiting

**Affiliations:** Department of Neurosurgery, Division of Functional Neurosurgery, Allegheny General Hospital, 320 E. North Avenue, Suite 302, Pittsburgh, PA 15212, USA

## Abstract

Obesity remains a pervasive global health problem. While there are a number of nonsurgical and surgical options for treatment, the incidence of obesity continues to increase at an alarming rate. The inability to curtail the growing rise of the obesity epidemic may be related to a combination of increased food availability and palatability. Research into feeding behavior has yielded a number of insights into the homeostatic and reward mechanisms that govern feeding. However, there remains a gap between laboratory investigations of feeding physiology in animals and translation into meaningful treatment options for humans. In addition, laboratory investigation may not be able to recapitulate all aspects of human food consumption. In a landmark pilot study of deep brain stimulation (DBS) of the lateral hypothalamic area for obesity, we found that there was an increase in resting metabolic rate as well as a decreased urge to eat. In this review, the authors will review some of the work relating to feeding physiology and research surrounding two nodes involved in feeding homeostasis, nucleus accumbens (NAc) and hypothalamus, and use this to provide a framework for future investigations of DBS as a viable therapeutic modality for obesity.

## 1. Introduction

Obesity is a pervasive global health problem [1, 2]. The World Health Organization (WHO) estimates that nearly 500 million people worldwide are obese [3]. A plethora of weight loss solutions exist, including gastric bypass surgery, but none have emerged as a durable solution. Surgical options for obesity include gastric bypass and banding surgery, which attempt to modulate the physiology of the gastrointestinal (GI) system to produce weight loss [4, 5]. However, as evidence indicates, much of the initial weight loss is regained and long-term complications [6, 7] from manipulation of the GI tract have made it less attractive in recent years. Scientific inquiry in recent years has found that obesity involves a complex interplay of neural networks that contribute to feeding behavior. While some argue that obesity is simply an imbalance between energy expenditure and food intake, evidence continues to mount that obesity may be the result of preserved feeding patterns that evolved in our ancestors due to inconsistent food availability. These behavioral patterns are particularly vulnerable to the ready availability of food and increased food palatability, both of which contribute to the growing epidemic of obesity. The failure of surgical modification of the GI tract to “cure” the epidemic of obesity may be a result of not addressing the underlying neurophysiologic basis of the disease. Feeding behavior then may be an interplay between a physiologic need for food and the reward system that powerfully motivates excessive eating in some individuals [8, 9].

Deep brain stimulation (DBS) has emerged as a minimally invasive, reversible method of neuromodulation first approved for movement disorders with expanded applications to a spectrum of neuropsychiatric disease including major depressive disorder (MDD) [10, 11], obsessive-compulsive disorder (OCD) [12–14], Tourette's syndrome (TS) [15–17], and addiction [18]. Insights gained from these studies, coupled with abundant animal research, led us to conduct the first human trial of DBS of the lateral hypothalamic area (LHA) for refractory morbid obesity [19]. In this paper, the authors will review some of the work relating to feeding physiology and research surrounding two nodes involved in feeding homeostasis, nucleus accumbens (NAc) and hypothalamus, and use this to provide a framework for future investigations of DBS as a viable therapeutic modality for obesity.

## 2. Physiologic Regulation of Feeding

Short- (cholecystokinin (CCK) and ghrelin) [20–22] and long-term (leptin, insulin) [20–22] satiety signals physiologically signal a nutrient abundance or deficit and regulate feeding behavior. Gastric distention by food intake causes the release of CCK, which acts on the nucleus of the solitary tract (NTS), which integrates taste and satiety information. Ghrelin release, in contrast, from the stomach peaks shortly before meal initiation and levels are found to rise after weight loss and may contribute to weight regain [23]. The discovery of leptin as a circulating satiety signal led to investigation in several human trials but disappointing results and the discovery that leptin resistance is common amongst obese individuals curtailed enthusiasm for its use [24]. Leptin is a small peptide that traverses the blood-brain barrier and acts on the arcuate nucleus of the hypothalamus, LHA, and the NTS. Though elevated leptin is a physiologic marker for adequate long-term energy stores, it also regulates feeding behavior during meals by augmenting the satiety response to CCK [25, 26].

Lesioning studies, first by Hetherington and Ranson [27] and later by Anand and Brobeck [28], paved way to the “classic” teaching of hypothalamic control of feeding behavior by two competing systems, one in the LHA and the other in the ventromedial hypothalamus (VMH). Lesions of the LHA resulted in cessation of feeding behavior and severe anorexia, while those of the VMH resulted in hyperphagia and obesity. Scientific inquiry in the decades following these landmark studies has revealed that feeding physiology cannot be distilled into a simple binary system of “on” and “off.” Instead, there appear to be complex interactions between clusters of hypothalamic nuclei that are powerfully governed by long-term hormonal signals such as leptin and insulin [29, 30]. Both hormones act on the arcuate nucleus of the hypothalamus, located inferolaterally to the walls of the third ventricle as well as the lateral hypothalamic area.

**Figure 1:**
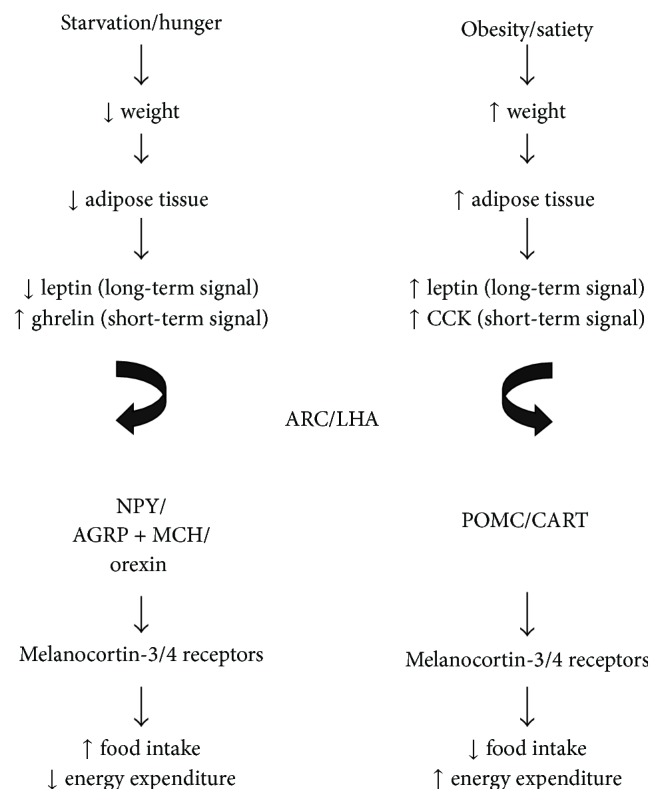
Starvation and satiety have opposing effects on levels of long-term (leptin, insulin) and short-term (ghrelin, CCK) signals that act on distinct neuronal populations within the hypothalamus. The effects of these neurons are mediated by melanocortin receptors and ultimately lead to increased or decreased intake based on the organism's needs.

The arcuate nucleus contains two distinct subpopulations of neurons: those expressing neuropeptide Y (NPY) and agouti-related peptide (AGRP) as well as those expressing proopiomelanocortin (POMC) and cocaine and amphetamine regulated transcript (CART) [31, 32]. The LHA contains neurons that produce melanin concentrating hormone (MCH) [33, 34] and orexins [35, 36]. Feeding behavior is promoted by NPY/AGRP and MCH/orexin neurons in the arcuate and LHA nuclei, respectively, while satiety is mediated by POMC/CART neurons [20, 37]. The physiological effects of melanocortin peptides are mediated by binding to melanocortin receptors, of which the melanocortin-3 and melanocortin-4 (MC3R and MC4R) subtypes are highly expressed in the central nervous system. In contrast to the agonist activity of melanocortins on the MC3 and MC4 receptors, NPY and AGRP are antagonists for these same receptor subtypes and thereby exert opposing physiological effects. In addition, NPY neurons have synaptic contacts on POMC neurons and have a net inhibitory effect and thus promote feeding [30, 31]. Obesity in mouse models can be generated by deletions or mutations of either the POMC gene [38] or MC3R/MC4R [39, 40] genes, and a similar phenotype is seen in humans that have deficiency of the MC4R [41, 42]. This evidence substantiates the importance of the melanocortin peptide and its associated receptors on energy homeostasis. Furthermore, leptin receptors are found on both POMC and NPY neurons, with the net effect of inhibiting NPY neurons and activating POMC neurons, thus resulting in satiety [30, 31]. In support of leptin's modulatory role on these distinct neuronal populations are mouse models that show that the obesity syndrome classically studied in leptin-deficient mice (Lep_ob/ob_), when crossed with NPY-null mice, reduces obesity as compared with Lep_ob/ob_ mice alone [43]. In a physiologically normal system, the fall in circulating leptin following weight loss decreases its inhibitory effect on the NPY/AGRP and LHA neurons and promotes feeding behavior (Figure 1) [20, 37]. In leptin-deficient mice, the downstream targets (NPY/AGRP neurons) are therefore constitutively active promoting hyperphagia and resulting in obesity. In contrast, VMH lesions tend to destroy the downstream targets of leptin action and leave the actions of the LHA unopposed, generating obesity.

### 2.1. Human Studies of DBS in the Hypothalamus

Evidence from animal models and lesioning studies led to two studies in which the VMH was targeted in 1 patient for obesity [44] with no effect on weight loss, though vivid autobiographical memories were enhanced, presumably by forniceal activation. Confirming the untoward effects of VMH stimulation, Wilent et al. exemplified the adverse psychogenic manifestations associated with this region when panic-attacks were induced in a graded manner with electrical stimulation of the VMH [45]. These unwanted adverse effects have since waned interest in targeting the VMH for obesity.

In our FDA-approved pilot study of 3 patients with refractory morbid obesity (all of whom had failed gastric bypass), we were able to demonstrate increases in resting metabolic rate in 2 of 3 patients using monopolar stimulation. Interestingly, traditional programming parameters as used for DBS in movement disorders did not result in changes in metabolism [19]. Biochemical profiles of hormones involved in obesity including T4, T3, insulin growth factor (IGF), leptin, AGRP, ghrelin, and NPY were all measured at baseline and after LHA-DBS with no change following stimulation. The finding that there was no change in these hormonal markers implies two possibilities: the fact that DBS may work as a modulator independent of hormonal changes or the fact that long-term follow-up is needed to determine whether changes do occur. Long-term programming at the RMR-optimized settings resulted in a decreased urge to eat as well as increased subjective feelings of energy that resolved when the stimulator was turned off, even in a blinded fashion. These findings suggest that DBS for obesity may involve distinct neural networks that need further investigation to determine optimal stimulation settings. Neuropsychological scales were also administered to all 3 patients that measured binge eating, body image, and feelings of hunger. Binge eating was reduced in one patient, and importantly this same patient reported reduced feelings of hunger. The two remaining patients while not showing any changes in binge eating behavior or feelings of hunger did show improvements regarding body image. While the weight lost by our patients was not significant, our pilot study was able to confirm the safety of LHA stimulation with few adverse events, laying groundwork for future studies.

## 3. Reward Integration of Feeding

Our work, in addition to those of others, has shown that combating obesity is more complex than simply changing feeding behavior but may also require modulation of reward networks. Leptin and insulin, in addition to modulating hypothalamic regions, have also been found to exert influences on reward circuitry such as the ventral tegmental area (VTA) [37, 46–48]. The VTA, located in the midbrain, provides much dopaminergic input to the nucleus accumbens (NAc), striatum, and other brain areas and is known to have receptors for leptin and insulin. As with hypothalamic areas, leptin and insulin appear to tonically inhibit the VTA, as demonstrated by experiments in which centrally administered leptin decreases sucrose preference [47], and it also decreases firing of neurons in the VTA [48]. In addition, the reward value of drugs has been shown to increase in states of food deprivation, confirming leptin's inhibitory role in the reward system [49–51]. Leptin circulates in proportion to body fat mass, and levels fall in states of food deprivation. This drop in leptin (see above) may release feeding centers (ARC/LHA) and the VTA from tonic inhibition and coupled with a compensatory rise in ghrelin may prime feeding behavior to restore lost weight.

### 3.1. Dopamine's Role in Reward

Examining the role of dopamine neurotransmission is essential for understanding reward integration of feeding behavior. Some researchers have also used neuroimaging studies to suggest that reward hypofunction is central to the pathophysiology of obesity as demonstrated by decreased D2 receptor availability in obese individuals [9]. Proponents of this hypothesis suggest that obesity may stem from a compensatory drive by individuals to “restore” normal levels of pleasure by overeating [52, 53]. However, abundant animal research into dopamine's function in the last two decades has revealed that it may not necessarily be responsible for the hedonic impact of stimuli and that there may exist a distinct but important dissociation between “liking” and “wanting” [8, 9, 54]. The dissociation of “liking” (hedonic experience) and “wanting” (desiring a stimulus) may be important in obese individuals, who may overeat because of a* desire* or* craving* that is out of conscious control than enjoying the hedonic component of eating. Obesity may therefore have similar pathophysiology to drug addiction, a view endorsed by some.

Dopamine has long been touted as the “pleasure” neurotransmitter [55] and that it is necessary for subjective feelings of hedonia. Animal experiments have shown that pharmacologic silencing (using 6-OHDA) of up to 99% of DA in the NAc and striatum failed to decrease “liking” responses to sweet rewards [54, 56]. Furthermore, experiments in mice where extracellular DA was artificially increased through knockdown of the dopamine transporter (DAT) failed to increase “liking,” though “wanting” (food-seeking behavior) was increased [57, 58]. This evidence suggests that dopamine may not be necessary to mediate feelings of reward [8, 9] in sharp contrast to other experiments that have shown that opioid, cannabinoid, and benzodiazepine administration demonstrate increased “liking” reactions to sweet reward [59–64]. The dissociation between hedonia and DA neurotransmission is also evident in patients with Parkinson's disease (PD), who have subjective hedonic experiences similar to control patients [65]. Other work has also shown that diet induced DA deficiency in healthy human subjects did not change subjective feelings of “liking” a cocaine reward though desire for cocaine seemed to wane [66]. Dopamine dysregulation in obese individuals may therefore be a consequence of the disease process and may not play a causal role in the development of obesity.

## 4. Hedonic Hotspots

The hedonic experience of food activates a number of brain structures that have reciprocal connections with each other. These structures include neocortical structures such as the orbitofrontal, anterior cingulate, and insular cortices, as well as phylogenetically older subcortical areas such as the NAc, ventral pallidum, amygdala, and parabrachial nucleus of the pons. Particularly compelling is that within these structures lie hedonic “hotspots” which amplify both desire (wanting) and hedonia (liking) for food. This evidence suggests that any modulation of feeding behavior (as in our study with the LHA) may not be as effective because it does not alter the* desire* for food. Two decades of animal research [8, 9, 54, 58, 59] has revealed that behavioral homologues exist between animal, primate, and human models where “liking” reactions are distinct from “disliking” or aversive reactions.

### 4.1. Nucleus Accumbens

The significant role of the nucleus accumbens in reward processing is undisputed. The NAc has been targeted in both animal and human studies, with the latter focusing on neuropsychiatric conditions such as MDD, OCD, and Tourette's as well as addiction [10–18]. In recent years, significant advances have shown that the NAc is made of two distinct regions: the core and the shell that subserve specific functions [67–69]. The shell of the accumbens receives afferents from the ventral medial prefrontal cortex as well as the VTA, areas that have been implicated in drug-seeking behavior [70, 71], while the core is innervated predominantly by the substantia nigra, involved with motor planning and execution [72]. These differences in functionality between the core and shell are recapitulated by their efferent projections, with the former projecting to premotor and supplementary motor cortices and the latter to subcortical motor areas, the LHA, and amygdala [69, 73, 74]. In rodent models, it was found that a small area in the rostrodorsal medial shell of the NAc by injection of opioid or endocannabinoid agonist significantly increased both “liking” (orofacial reactions) and wanting for food rewards [63, 64]. Surprising, however, was that opioid or endocannabinoid agonists into areas outside this “hotspot” increased wanting for food without changing the absolute number of liking orofacial reactions. This finding suggests that the dissociation between “liking” and “wanting” may be applicable to humans. In parallel to drug addicts, obese individuals may simply overeat not because they “like” food more, but simply “crave” it more than normal individuals when exposed to palatable food or food-related cues. It also highlights the importance of any future study targeting the NAc for obesity, as the goal is to modulate craving without changing the hedonic experience of food.

### 4.2. Ventral Pallidum

A hedonic hotspot has also been identified in a small area in the posterior part of the ventral pallidum and recent studies have shown that it is also intricately involved in the processing of food reward. Historical lesioning studies of the 1960s and 70s damaged the LHA, resulting in hypophagia, but also decreased “liking” reactions to sweet reward by damaging the ventral pallidum [8, 9, 75, 76]. Furthermore, as with the previously mentioned NAc hotspot, an opioid agonist injected increased the number of “liking” orofacial reactions while neuronal cell death or chemical inactivation (by GABA agonists) led to aversive reactions even for normally palatable sweet rewards [8, 9, 77]. It has also been found that pallidal neurons code for reward based on physiologic states. In normal conditions, pallidal neurons fire vigorously when sweet reward (sucrose) is administered but not to excessively salty water in rodents. However, the induction of salt appetite by administration of diuretics causes pallidal neurons to fire in a similar fashion to baseline firing for sweet reward [78, 79]. This is compelling evidence that the physiologic state of an organism is crucial in perception of whether a stimulus is perceived as pleasurable. Importantly, there also appear to be direct projections of orexin neurons [35, 36] to the pallidal hotspot, coupling feeding behavior directly with hedonic experience [80, 81]. Orexigenic neurons through direct projections to posterior ventral pallidum may provide incentive to seek food beyond physiologic need by acting on one node in the reward system. While evidence of these hotspots is compelling, it remains to be determined whether such homologous regions exist in humans. Research has also shown that in addition to modulating “liking” independently, the NAc and pallidum work together [82]. Injection of an opioid agonist into either NAc or pallidum activates the other structure and this dual activation may act synergistically to produce “liking.”

## 5. The Incentive Salience Theory

Developed by Berridge and Robinson et al. [8, 9], the incentive salience theory is an attempt to elucidate the true role of dopamine neurotransmission. At its essence, incentive salience is distinctly different from the hedonic experience of a reward such as food. According to Berridge and Robinson, incentive salience is the “active assignment of salience and attractiveness to visual, auditory, tactile, or olfactory stimuli that are themselves intrinsically neutral. Salience attribution possesses the qualities of wanting and desiring, but these need to be distinguished from the experience of sensory pleasure.” [8] Incentive salience then is the mechanism by which “wanting” a particular reward stimulus (food, sex, drugs, etc.) is generated. This is distinctly different from explicit or declarative “wanting” which is mediated at higher cortical levels [8, 9]. Most importantly, incentive salience is dynamic and highly dependent on the physiologic state of the organism and drives behavior subconsciously.

Central to the generation of incentive salience of a particular reward stimulus is mesolimbic dopaminergic neurotransmission. Particularly, palatable food may take advantage of normal neural mechanisms that evolved to reinforce feeding behavior when food was scarce to allow an organism to survive. The importance of incentive salience to understanding the pathophysiology of obesity is that it provides a link between the physiologic* need* to eat and the hedonic aspects of food consumption.

Berridge's three-stage model of incentive salience [8] is based on early Pavlovian experiments involving classical conditioning. In the first stage, a new stimulus (such as palatable food) is encountered by an organism and is intrinsically neutral. If the stimulus (i.e., taste) is perceived as pleasurable, it activates “liking” and secondarily activates “wanting.” The rewarding stimulus that predicted the hedonic experience is assigned incentive salience. Upon exposure to the rewarding stimulus subsequently (food), the incentive salience of whatever predicted it (contextual environment such the location, aroma of food, etc.) is potentiated. In the final stage, mesolimbic dopaminergic transmission occurs to generate incentive salience anew each time the rewarding stimulus is encountered and is modulated by physiologic states such as hunger to powerfully motivate behavior. Obese individuals may therefore not, as some have argued, overeat because of an increased sensory pleasure for food but because their levels of wanting food may be altered by years of chronic overconsumption.

## 6. Rationale for Neuromodulation

The abundance of research into obesity pathophysiology has indicated that it is a complex problem involving multiple brain regions. From an evolutionary perspective, feeding behavior is promoted by redundant neuronal systems, presumably to promote survival in times of food scarcity. As a result, while obesity can result from a number of monogenic alterations [38–42], human obesity is rarely caused by single gene mutations. Instead, increased food availability and palatability promote overeating long after metabolic demands are met. It is therefore unlikely that a single drug or brain region will “cure” all forms of obesity. In addition, animal models of obesity may not have face validity with human feeding behavior due to a lack of incorporation of a cortical control mechanism [21]. Animal models tend to emphasize palatability represented by the fat or carbohydrate content of chow, which on a superficial level may resemble the choices available to a human consumer. However, these models fail to incorporate other aspects of food-seeking behavior including context, variety, and previous exposure to a palatable stimulus (i.e., akin to drug seeking) as powerful influences desire and, ultimately, food consumption. In addition, the discovery of hedonic “hotspots” in animals wherein “liking” and “wanting” in the nucleus accumbens are dissociative properties suggests that increased food intake may occur independently of the pleasurable experience of food consumption [63, 64, 77–79].

The limitations of animal models combined with increased understanding that feeding physiology is complex and relies on input and integration from both homeostatic and hedonic neural circuits argue for further investigation. While neuromodulation in obesity is relatively new, its safety and efficacy profile in thousands of patients with a variety of movement and psychiatric disorders argues for further investigation. While our pilot study [19] did not yield weight loss, it resulted in a decreased urge to eat in all patients, confirming prior animal and human research that the LHA is a central node in the generation of feeding behavior. However, to simply relegate feeding behavior to an “on” or “off” state would be inaccurate as it is now understood that the control of feeding is powerfully regulated by physiologic states and reward valuation [8, 9]. Without adequately addressing the hedonic component of food seeking and the motivational processes that drive eating, any intervention is likely to be unsuccessful.

### 6.1. Future Perspectives

As demonstrated by our pilot study of LHA-DBS, neuromodulation for obesity is still in its infancy. Future studies of DBS in obesity should enroll a greater number of patients in order to discern the optimal stimulation parameters as well as identify individual patient characteristics that may contribute to the success or failure of DBS as a therapeutic modality for refractory obesity. Additionally, future studies of DBS in obesity should attempt to modulate the reward circuitry either independently or in conjunction with areas such as the LHA and ARC in order to target both essential components of feeding physiology. Arguments for targeting nodes in reward, such as the NAc, stem from the unintended side effects of DBS for other conditions such as OCD in which one patient experienced smoking cessation and another abstinence from alcohol after years of dependence [83, 84]. While obesity and pathological overeating may have some similarities to drug addiction, it should be noted that they have distinct differences [21]. Nonetheless, both addiction and obesity may stem from reward system dysfunction and thus neuromodulation of reward centers may be beneficial in treating obesity.

As alluded to earlier, fMRI imaging has also been performed in obese patients and healthy controls with some suggesting that reward hypofunction may be central to obesity pathophysiology. Imaging studies, such as fMRI, may be used in conjunction with DBS over time to test this hypothesis and to determine whether there are functional differences in patients with obesity that change over time with neuromodulation of brain regions such as the ARC, LHA, and NAc. It is highly likely that DBS studies of the future may also shed light into distinct obesity subtypes that are not recognized today and may have led insight into which patients would benefit most from DBS.

### 6.2. Ethical Considerations

Opponents of neuromodulation for obesity may argue that it is unethical because it may alter behavior and therefore be compared to psychosurgery. However, in contrast to the ablative nature of the psychosurgical procedures of the past, DBS is slowly becoming an accepted treatment modality for those with intractable psychiatric disease including MDD, OCD, Tourette's, and even addiction. The safety of our pilot study of LHA-DBS and studies for psychiatric disease in which the nucleus accumbens were targeted suggests that neuromodulation is a worthwhile endeavor to better delineate the mechanisms that may be involved in pathological overeating. Investigation into obesity may yield insight into other conditions that have aberrant feeding physiology such as anorexia and bulimia and also may pave the way for expanded applications into disorders such as addiction.

## 7. Conclusion

In the last several decades, the understanding of feeding physiology has grown considerably more complex. But while the neural networks that govern feeding have become clearer, treatment of pathological overeating that can lead to obesity and obesity-related comorbidities has stalled. In addition, the surgical options (gastric bypass, banding) are available to patients when conservative measures are not without adverse effects. The safety, reliability, of DBS in movement and neuropsychiatric disease encourages investigation in obesity as a potential therapeutic option in patients of whom other forms of treatment have failed.
